# Supplementary material for: Mapping the intersection of social status and comorbidity in knee osteoarthritis: A WOMAC-based study
Source: PLoS One. 2026 Feb 13;21(2):e0324767. doi: 10.1371/journal.pone.0324767 (PMC12904383; doi:10.1371/journal.pone.0324767)
Supplement: S2 Appendix — The included questions comprised the questionnaire administered to the participants. (DOCX) [file pone.0324767.s002.docx]

**QUESTIONNAIRE**

Serial No……………………………………. Date………………………………….

Name……………………………...

| **Variables** | **Categories** | **Code** |
| --- | --- | --- |
| **Socio-demographic characteristics:** | | |
| 1. Age | 1. 60 to 69 years 2. 70 and above years | 1  2 |
| 1. Gender | 1. Male 2. Female | 1  2 |
| 1. Marital status | 1. Married 2. Unmarried | 1  2 |
| 1. Education | 1. Illiterate 2. Primary 3. Secondary and above | 1  2  3 |
| 1. Monthly family income | 1. 25,000 and below 2. 25,001 to 49,999 3. 50,000 and above | 1  2  3 |
| 1. Financial independence | 1. Independent 2. Dependent | 1  2 |
| 1. **Disease related factors:** | | |
| 7. Diabetes | 1. No 2. Yes | 1  2 |
| 8. Obesity | 1. No 2. Yes | 1  2 |
| 9. Cardiovascular disease | 1. No 2. Yes | 1  2 |

**Scoring of Osteoarthritis (WOMAC Scale)**

| **Rate your pain when** | **None** | **Slight** | **Moderate** | **Severe** | **Extreme** | **Investigator**  **use only** |
| --- | --- | --- | --- | --- | --- | --- |
| Walking | 0 | 1 | 2 | 3 | 4 |  |
| Climbing stairs | 0 | 1 | 2 | 3 | 4 |  |
| Sleeping at night | 0 | 1 | 2 | 3 | 4 |  |
| Resting | 0 | 1 | 2 | 3 | 4 |  |
| Standing | 0 | 1 | 2 | 3 | 4 |  |
| **Rate your stiffness in the…..** |  |  |  |  |  |  |
| Morning | 0 | 1 | 2 | 3 | 4 |  |
| Evening | 0 | 1 | 2 | 3 | 4 |  |
| **Rate your difficulty when….** |  |  |  |  |  |  |
| Descending Stairs | 0 | 1 | 2 | 3 | 4 |  |
| Ascending stairs | 0 | 1 | 2 | 3 | 4 |  |
| Rising from sitting | 0 | 1 | 2 | 3 | 4 |  |
| Standing | 0 | 1 | 2 | 3 | 4 |  |
| Bending to floor | 0 | 1 | 2 | 3 | 4 |  |
| Walking on even floor | 0 | 1 | 2 | 3 | 4 |  |
| Getting in/out of car | 0 | 1 | 2 | 3 | 4 |  |
| Going shopping | 0 | 1 | 2 | 3 | 4 |  |
| Putting on socks | 0 | 1 | 2 | 3 | 4 |  |
| Rising from bed | 0 | 1 | 2 | 3 | 4 |  |
| Taking off socks | 0 | 1 | 2 | 3 | 4 |  |
| Lying in bed | 0 | 1 | 2 | 3 | 4 |  |
| Getting in/out bath | 0 | 1 | 2 | 3 | 4 |  |
| Sitting | 0 | 1 | 2 | 3 | 4 |  |
| Getting in/out toilet | 0 | 1 | 2 | 3 | 4 |  |
| Doing light domestic duties (cooking, dusting) | 0 | 1 | 2 | 3 | 4 |  |
| Doing heavy domestic duties (moving furniture) | 0 | 1 | 2 | 3 | 4 |  |
| **Total score (out of 96)** | | | | | |  |
